# Early Exposure to THC Alters M-Cell Development in Zebrafish Embryos

**DOI:** 10.3390/biomedicines8010005

**Published:** 2020-01-04

**Authors:** Md Ruhul Amin, Kazi T. Ahmed, Declan W. Ali

**Affiliations:** 1 Department of Biological Sciences, CW-405 Biological Sciences Bldg., University of Alberta, Edmonton, AB T6G 2E9, Canada; mdruhul@ualberta.ca (M.R.A.); ktahmed@ualberta.ca (K.T.A.); 2 Neuroscience and Mental Health Institute, University of Alberta, Edmonton, AB T6G 2E1, Canada

**Keywords:** cannabinoids, Mauthner, motor neurons, muscle, NMJ, CNS

## Abstract

Cannabis is one of the most commonly used illicit recreational drugs that is often taken for medicinal purposes. The psychoactive ingredient in cannabis is Δ^9^-Tetrahydrocannabinol (Δ^9^-THC, hereafter referred to as THC), which is an agonist at the endocannabinoid receptors CB_1_R and CB_2_R. Here, we exposed zebrafish embryos to THC during the gastrulation phase to determine the long-term effects during development. We specifically focused on reticulospinal neurons known as the Mauthner cells (M-cell) that are involved in escape response movements. The M- cells are born during gastrulation, thus allowing us to examine neuronal morphology of neurons born during the time of exposure. After the exposure, embryos were allowed to develop normally and were examined at two days post-fertilization for M-cell morphology and escape responses. THC treated embryos exhibited subtle alterations in M-cell axon diameter and small changes in escape response dynamics to touch. Because escape responses were altered, we also examined muscle fiber development. The fluorescent labelling of red and white muscle fibers showed that while muscles were largely intact, the fibers were slightly disorganized with subtle but significant changes in the pattern of expression of nicotinic acetylcholine receptors. However, there were no overt changes in the expression of nicotinic receptor subunit mRNA ascertained by qPCR. Embryos were allowed to further develop until 5 dpf, when they were examined for overall levels of movement. Animals exposed to THC during gastrulation exhibited reduced activity compared with vehicle controls. Together, these findings indicate that zebrafish exposed to THC during the gastrula phase exhibit small changes in neuronal and muscle morphology that may impact behavior and locomotion.

## 1. Introduction

THC (Δ^9^-Tetrahydrocannabinol) is the main psychotropic ingredient in the plant *Cannabis sativa*. THC binds to and activates two distinct classes of G-protein coupled receptors: cannabinoid receptors 1 (CB_1_R) and cannabinoid receptors 2 (CB_2_R) [1]. CB_1_Rs are localized to the central nervous system (CNS) [2,3,4], whereas CB_2_Rs are mainly associated with the peripheral nervous system, the immune system [5,6], the digestive and reproductive systems, and to a small extent the CNS [7,8,9]. In chicks and mice, CB_1_R protein expression occurs even before the onset of neuronal development [10] and increases in a location-specific manner [11]. In rats, the offspring of mothers that were exposed to THC during gestation show different locomotor and exploratory behavior compared with controls [12], and in humans, prenatal exposure to THC leads to increased incidences of tremors and startle behaviors [13]. Significant evidence has been accumulated to show that prenatal or embryonic exposure to cannabinoids alters a range of behaviors, physiological processes, and gene expression, in large part because it appears to affect the normal functioning of the endocannabinoid (eCB) system. With regard to CNS development, the eCB system has been shown to regulate neural progenitor proliferation, specification, and migration (Reviewed in [14]), axonal growth, pathfinding and fasciculation [3,15], and the development of appropriate synaptic activity [16].

In zebrafish, CB_1_Rs are highly expressed in the hindbrain where they are associated with reticulospinal neurons [3]. In fact, zebrafish express both CB_1_Rs and CB_2_Rs in the embryonic stages of development [17]. CB_1_R expression appears low in early development prior to 24 hpf but increases as development proceeds, whereas CB_2_R expression follows the reverse pattern, with high levels prior to 24 hpf and lower relative levels thereafter [17]. The knockdown of CB_1_R expression with morpholino antisense oligonucleotides, or block of CB_1_Rs with the receptor blocker AM251 alters patterns of axonal growth [3]. These findings prompted us to ask whether early exposure to THC alters the development of the primary reticulospinal neurons in the zebrafish hindbrain, the Mauthner cell (M-cell). We specifically focused on M-cell morphology and aspects of locomotion associated with M-cell function, such as the escape response to touch. M-cell neurons first appear around 8–9 hours post fertilization (hpf) in the middle of the developmental period known as gastrulation. In zebrafish, gastrulation occurs from 5.25 hpf to 10.75 hpf [18]. At this stage, three germ layers are formed (ectoderm, mesoderm, and endoderm) and primary neurons, including M-cells appear. Shortly after their birth, the M-cells project an axon contralaterally and caudally down the spinal cord to the tail region [19]. As each M-cell projects down the cord, it forms synapses with primary motor neurons which innervate the white muscle fibers of the trunk [19].

We had previously found that zebrafish embryos exposed to THC during gastrulation exhibited altered fast escapes in response to acoustic but not mechanosensitive stimuli [20], indicating a possible deficit with M-cell form or function. Our results from the present study indicate that M-cells are largely intact following exposure to THC during gastrulation and that there appears to be minor but significant changes to neuronal morphology. Moreover, muscle morphology and locomotor responses are also impacted by exposure to THC.

## 2. Experimental Section

### 2.1. Animal Care and Exposure to THC

The fish used in this study were wild type zebrafish (*Danio rerio*) embryos of the Tubingen Longfin (TL) strain that were maintained at the University of Alberta Aquatic Facility. All animal housing and experimental procedures in this study were approved by the Animal Care and Use Committee at the University of Alberta (AUP #00000816) and adhered to the Canadian Council on Animal Care guidelines for humane animal use. For breeding, 3–5 adults, usually consisting of 3 females and 2 males, were placed in breeding tanks the evening before eggs were required. The following morning, fertilized eggs were collected from the breeding tanks, usually within 30 min of fertilization. Embryos and larvae were housed in incubators on a 12 h light/dark cycle, and set at 28.5 °C. Embryos were exposed to egg water (EW; 60 mg/mL Instant Ocean) containing either 6 mg/L THC (diluted from a stock solution obtained from Sigma; Δ^9^-Tetrahydrocannabinol solution 1.0 mg/mL in methanol) or equivalent amounts of methanol during the period of gastrulation, which occurs between 5.25 hpf and 10.75 hpf. The exposure medium was then replaced at 10.75 hpf with 25 mL of fresh EW. Embryos were washed several times in EW and then incubated in fresh EW until further experiments at 48 hpf. For immunohistochemical studies, pigment formation was blocked by adding 0.003% phenylthiourea (PTU) dissolved in egg water at 24 hpf. The dose of THC (6 mg/L) was selected based on our previous work identifying critical concentration that affects survival and embryonic development [20].

### 2.2. Immunohistochemistry

Embryos (2 dpf) were fixed in 2% paraformaldehyde for 1–2 h and washed with 0.1 M phosphate buffered saline (PBS) every 15 min for 2 h. The preparations were then permeabilized for 30 min in 4% Triton-X 100 containing 2% BSA and 10% goat serum. Tissues were incubated for 48 h at 4 °C in either mouse monoclonal anti-3A10 (Developmental Studies Hybridoma Bank, Iowa City, IA, USA) (1:250) which targets neurofilaments associated with M-cell [21] or anti-RMO44 (Thermo Fisher Scientific, Waltham, MA, USA) (1:250) which labels several types of reticulospinal neurons. Tissues were also incubated in anti-F59 which targets myosin heavy chain (Developmental Studies Hybridoma Bank, 1:50) isoform of red muscle fibers [22] or anti-F310 (Developmental Studies Hybridoma Bank, 1:100) that targets myosin light chain 1 and 3f of white muscle fibers [23]. Tissues were washed in PBS twice every 15 min for 2–3 h and then incubated for 4 h at room temperature in the secondary antibody, Alexa Fluor® 488 goat anti-mouse IgG or Alexa Fluor® 555 goat anti-mouse IgG, (Molecular Probes, Thermo Fisher Scientific), at a dilution of 1:1000. The embryos were then washed for 7 h with PBS and mounted in MOWIOL mounting media. For the labelling of nicotinic acetylcholine receptors (nAChRs), embryos at 2 dpf were permeabilized as previously stated and incubated with 100 nM Alexa-488 conjugated α-bungarotoxin (Molecular Probes, Thermo Fisher Scientific) for 4 h at room temperature. Embryos were then washed for 7 h with PBS and mounted in MOWIOL mounting media. All embryos were imaged on a Zeiss LSM 710 confocal microscope (CA, USA) and photographed under a 40x objective. Images were compiled using Zeiss LSM Image Browser software and are shown as maximum intensity z-stack compilations. Measurements of the images were done using Image J (ImageJ 1.51r, National Institutes of Health, Bethesda, MD, USA).

### 2.3. Escape Response in 2 dpf Embryos

Escape responses of 2 dpf embryos were tested and recorded as previously described [24]. Briefly, 2 dpf embryos were immobilized in 2% low-melting point agarose (LMPA; Sigma-Aldrich; St. Louis, MO, USA) dissolved in embryo medium. LMPA was cut away from the embryo’s trunk and tails allowing them to move, while the heads remained embedded in the gel. Embryo media was added to the petri dish to ensure that the embryos remained immersed in solution. Borosilicate glass micropipettes were pulled, filled with solution and then positioned close to embryo’s otolith without touching the embryo. Embryos were stimulated using a 15 ms pulse of phenol red (Sigma-Aldrich) dissolved in embryo media ejected from a Picospritzer II (General Valve Corporation, Cambridge, MA, USA). Embryonic responses were recorded for about 900 ms following the stimulus using an AOS video camera (AOS S-PRI 1995; 1250 FPS; shutter speed: 800 μs) mounted on a dissecting microscope. The video-recordings were analyzed using a Motion Analysis Software, ProAnalyst® (Xcitex Inc., Cambridge, MA, USA).

### 2.4. qPCR of nAChR Subunits

To analyze the expression of different nAChR subunits, mRNA was extracted from whole embryos (*n* = 30–50 embryos, *N* = 5 batches) using a Trizol reagent according to manufacturer protocol. The concentration and purity of the RNA was determined by NanoDrop spectrophotometry (Thermo Fisher Scientific). A Maxima First Strand cDNA Synthesis kit (Thermo Fisher Scientific) was used to synthesize cDNA from 1 μg of the mRNA stocks according to the manufacturer’s protocol. cDNA was diluted to 1:40 in 1 × TE buffer for real-time PCR reaction. TaqMan gene expression assays (Thermo Fisher Scientific) for zebrafish *chrna*1, *chrng* and *chrne* that were previously validated [25] were reused for qPCR reaction.

Quantitative real-time PCR was carried out with the 7500 Fast system (Applied Biosystems). For each reaction (10 μL), 5 μL of 2 × TaqMan Gene Expression Mastermix, 0.5 μL of 20 × TaqMan Gene Expression Assay, and 2.5 μL of Nuclease-free water was added to 2 μL of cDNA diluted to 1:40. The thermal profile included a holding step of 50 °C for 2 min followed by another holding step of 95 °C for 10 min, and 40 cycles including denature at 95 °C for 5 s and anneal/extend at 60 °C for 1 min. All samples were run in triplicate and the threshold cycle (Ct) was determined automatically by SDS software (Applied Biosystems). Outliers possibly originating from inaccurate pipetting were omitted and Ct values were averaged. Housekeeping gene Beta -actin (*actb*1) was used as internal control for our calculation. Comparative Ct Method (DDCt) was used for data representation using vehicle control as calibrator. No template controls (NTC) were included for each assay in every plate as negative control.

### 2.5. Locomotor Activity in 5 dpf Larva

To track locomotor activities, individual 5 dpf larvae were placed in a single well of a 96-well plate, then video-taped, and the data analysed according to previously published procedures [26,27]. Larvae were gently positioned in the centre of wells containing 150 µL egg water, pH 7.0 and 48 wells were used each time from a 96 well plate in our study (Costar #3599). Prior to video recording, larvae were acclimated in the well plate for 60 min. Plates were placed on top of an infrared backlight source and a Basler GenlCaM (Basler acA 1300-60) scanning camera with a 75 mm f2.8 C-mount lens, provided by Noldus (Wageningen, Netherlands) was used for individual larval movement tracking.

EthoVision ® XT-11.5 software (Noldus) was used to quantify activity (%), velocity (mm/s), swim bouts frequency and cumulative duration of swim bouts for one hour. To exclude background noise, ≥0.2 mm was defined as active movement. Activity was defined as % pixel change within a corresponding well between samples (motion was captured by taking 25 samples/frames per second) as reported previously [27].

### 2.6. Statistics

All values are reported as means ± SEM (standard error of the mean). Significance was determined using a non-parametric *t*-test between vehicle and treated group followed by Mann-Whitney analysis where appropriate (*p* < 0.05). Comparisons between multiple groups were done by one-way ANOVA followed by a Tukey post-hoc multiple comparisons test. Statistical analysis was done using the statistical software built in to GraphPad prism.

## 3. Results

### 3.1. THC Exposure Reduces Axonal Diameter of M-Cell

**Figure 1:**
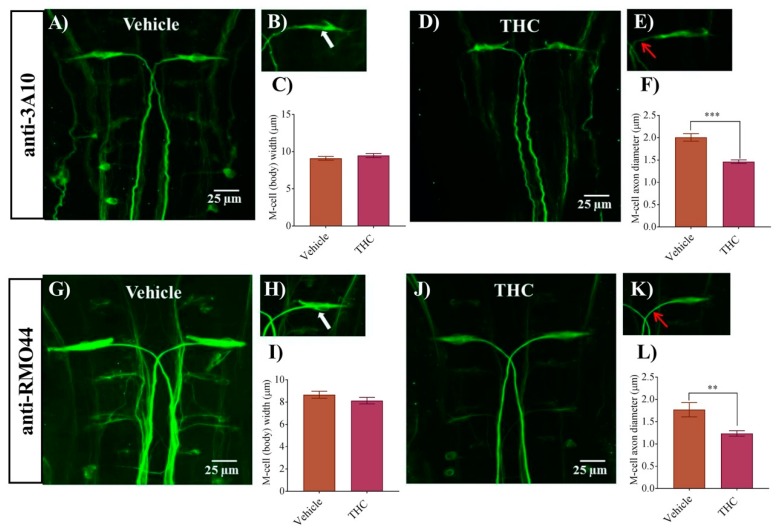
Δ^9^-Tetrahydrocannabinol (THC) exposure reduces M-cell axonal diameter. (**A**,**G**) Immunolabeling of M-cells with anti-3A10 and anti-RMO44 in a vehicle-treated embryo; (**B**,**H**) Higher magnification of M-cell body and axon of vehicle-treated embryos. White arrow shows the cell body of the M-cell. (**C**,**I**) Bar graph of the width of an M-cell body in vehicle and THC treated embryos. (**D**,**J**) Immunolabeling of M-cells with anti-3A10 and anti-RMO44 in a THC-treated (6 mg/L) embryo; (**E**,**K**) Higher magnification of M-cell body and axon of a THC-treated embryo. Red arrow points to the proximal axon immediately anterior to the decussation point. (**F**,**L**) Bar graph of the diameter of M-cell axons slightly anterior to the decussation point in vehicle and THC-treated embryos. ** Significantly different from vehicle control, *p* < 0.005. *** significantly different from vehicle control, *p* < 0.001.

In a previous study, we found that zebrafish embryos exposed to THC from 1–10 mg/L exhibited morphological and neuronal changes that ranged from no effect at the lower concentrations, to disorganized neuronal morphology and alterations in responses to sound at the higher concentrations [20]. In the present study we continue our work by examining the morphology of M-cells following exposure to the primary psychoactive ingredient in cannabis, THC. We exposed zebrafish embryos to 6 mg/L THC as we had done previously and compared these embryos with vehicle controls (0.6% methanol). An immunohistochemical analysis of M-cell morphology was performed at 2 dpf with anti-3A10. Embryos exposed to THC exhibited M-cells that were largely similar to controls but appeared disheveled and possessed slightly thinner and wispier looking axons (Figure 1A–E). The diameter of the M-cell body was unchanged (*p* > 0.05; *n* = 8–10) (Figure 1C), whereas the M-cell axon diameter was significantly smaller in the treated group compared with controls (*p* < 0.05). Specifically, the M-cell diameter in the control group was 2.0 ± 0.1 µm (*n* = 8) while it was 1.5 ± 0.1 µm (*n* = 11) in the THC treated group (Figure 1F). To confirm these findings, we performed an additional immunohistochemical analysis of the M-cells by labelling reticulospinal neurons using the anti-RMO44 antibody. We found that there was an overall reduction in the intensity of the fluorescent labelling of many neurons in the THC-treated animals compared with controls (Figure 1G,J). The diameter of the M-cell body remained unchanged (Figure 1I); however, the diameter of the M-cell axon was significantly smaller (1.2 ± 0.06 µm, *n* = 9) in the treated group compared with vehicle controls (1.8 ± 0.1 µm, *n* = 7) (*p* < 0.05) (Figure 1L). These results, obtained using two distinct and independent antibodies, strongly suggest that the M-cells exhibit small but significant changes following exposure to 6 mg/L THC in the gastrulation stage. 

### 3.2. Escape Response Properties Were Altered Due to THC Exposure

**Figure 2:**
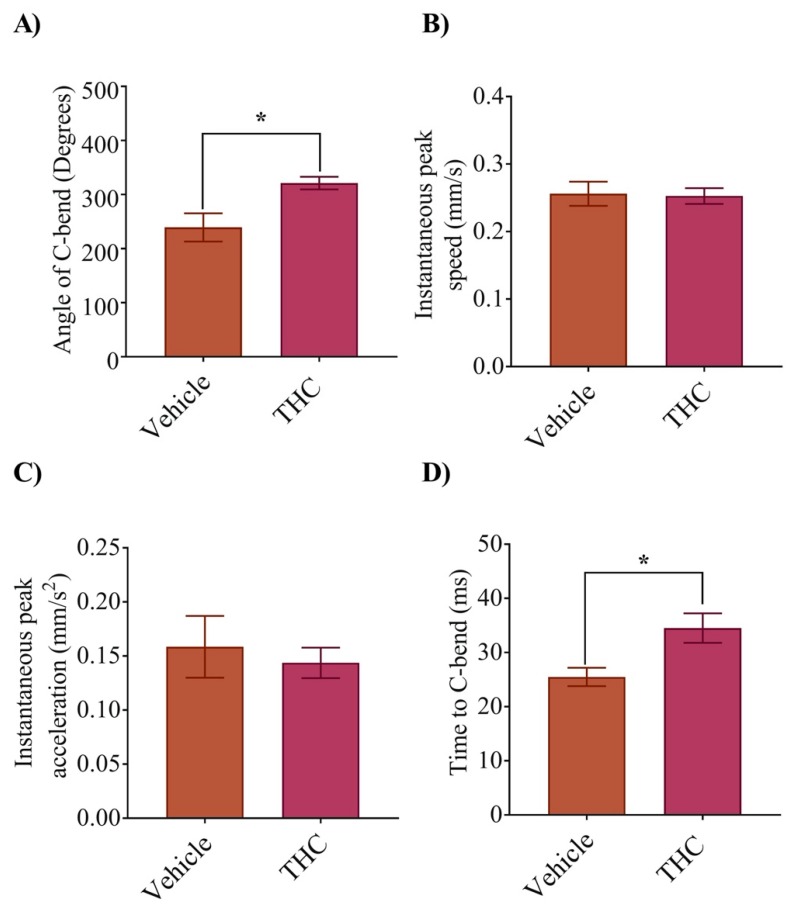
Exposure to THC during gastrulation alters escape response parameters. Analysis and quantification of C-bend parameters was carried out at 2 dpf. Zebrafish embryos exhibit a C-bend in response to a jet of water directed at the head just behind the eyes. (**A**) Bar graph shows the maximum angle of bend for vehicle and THC-treated (6 mg/L) embryos. (**B**) Shows the instantaneous peak speed (mm/s) during c-bend. (**C**) Shows the instantaneous peak acceleration during C-bend. (**D**) Bar graph showing the time for the tail to bend to the maximum angle. * Significantly different from vehicle control, *p* < 0.05.

To determine if the properties of the escape response had been altered by exposure to THC, we recorded the C-bend following a mechanosensitive stimulus to the head of 2 dpf embryos. The C-bend response rate between the two groups was similar and there were no overt differences between the treatments. However, the angle of the C-bend was significantly greater in the THC treated animals compared with vehicle controls (Figure 2A; *p* < 0.05; *n* = 7–13). Analysis of the maximum speed and acceleration showed no significant differences in these parameters (Figure 2B,C; *p* > 0.05; *n* = 7–13). Further, the time to maximum bend of the trunk was greater in the THC treated animals (Figure 2D, *p* < 0.05; *n* = 7–13), likely because the bend angle was greater.

### 3.3. White and Red Muscle Fibers Appear Thinner and Slightly Disorganized in THC Treated Embryos

**Figure 3:**
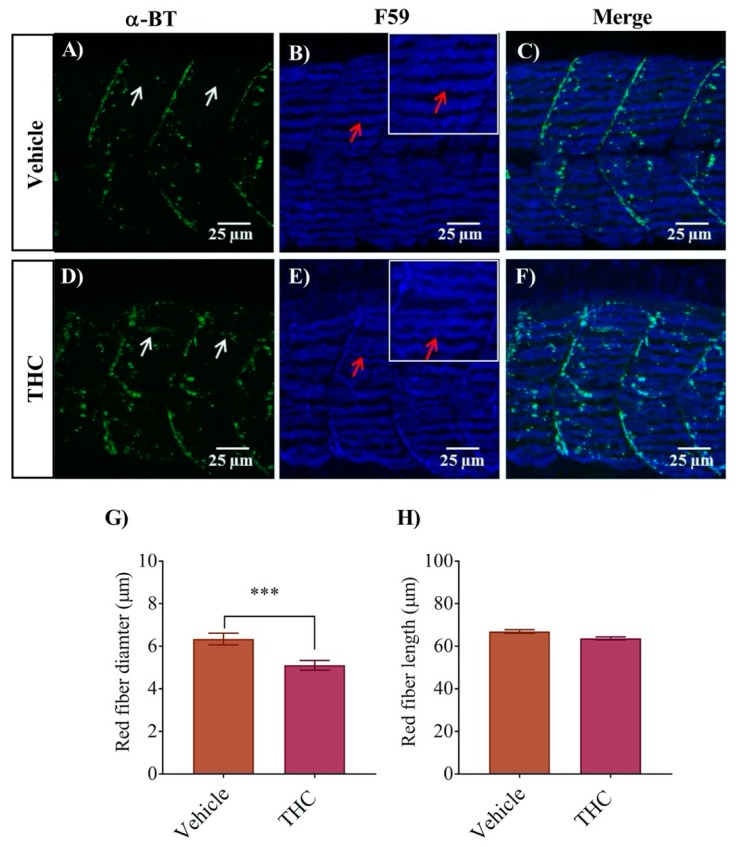
Co-labeling of red muscle fibers and nAChRs using anti-F59 and Alexa 488 conjugated α-bungarotoxin respectively. (**A**) α-Bungarotoxin labelled nAChRs associated with red muscle fibers in vehicle-treated embryos. (**B**) Anti-F-59 labelled muscle fibers. Red arrows point to the edge of a muscle fiber. Inset shows muscle fibers at higher magnification to better determine the size of the fiber. (**C**) Merged image showing the co-labeled red muscle fiber and nAChR in vehicle-treated animals. (**D**) α-bungarotoxin labelled nAChRs associated with red muscle fibers in THC-treated (6 mg/L) embryos. White arrow shows the cluster of nAChRs. (**E**) Anti-F59 labelled muscle fibers. Red arrows point to the edge of a muscle fiber. Inset shows muscle fibers at higher magnification to better determine the size of the fiber. (**F**) Merged image showing the co-labeled red muscle fiber and nAChR in THC-treated animals. (**G**) Bar graph showing the diameter of red fibers for vehicle and THC treated embryos and (**H**) Measurement of red fiber length. *** significantly different from vehicle control, *p* < 0.001.

To determine if the small changes in the C-bend escape response could be accounted for by properties of the muscle fibers, we performed an immunohistochemical analysis of the trunk muscles in conjunction with labelling of the nicotinic receptors using fluorescently tagged α- bungarotoxin. The trunk muscles of embryonic and larval zebrafish embryos are composed of a single layer of outer red muscles and several layers of inner white muscles [28]. The outer red muscle of vehicle control animals developed in an orderly fashion with clear and precise boundaries between the trunk segments (Figure 3). The α-bungarotoxin labelling of nAChRs in untreated animals was neatly aligned at the segmental boundaries (Figure 3A–C) as described in previous studies [29,30]. However, embryos treated with THC exhibited thinner individual muscle fibers (Figure 3E) that appeared less tightly packed, with larger spaces in between the fibers and unclear segmental boundaries. The diameter of THC treated red muscle fiber was reduced to 5.1 ± 0.2 µm from control values of 6.3 ± 0.3 µm in vehicle exposed fibers (Figure 3G, *p* < 0.05; *n* = 24–34). However, the lengths of the fibers remained unchanged (Figure 3H). Moreover, the nAChR expression, that was largely confined to the segmental boundaries, was somewhat disorganized (Figure 3D–F).

**Figure 4:**
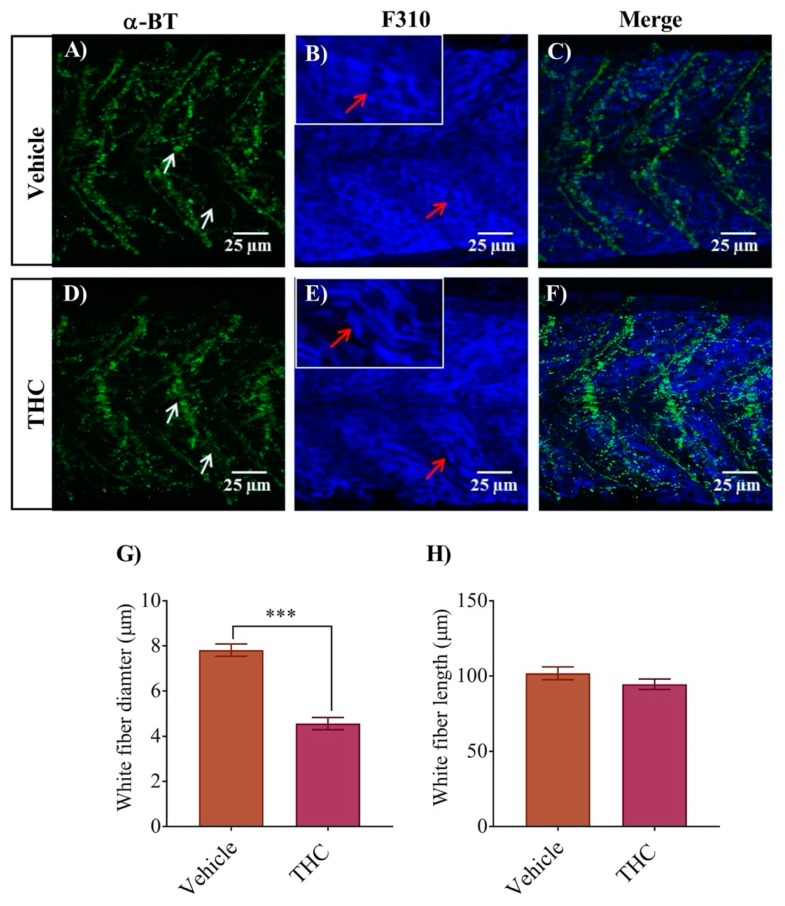
Co-labeling of white muscle fibers and nAChRs using anti-F310 and Alexa 488 conjugated α-bungarotoxin respectively. (**A**) α-Bungarotoxin labelled nAChRs associated with white muscle fibers in vehicle-treated embryos. White arrow shows clusters of nAChRs. (**B**) Anti-F-59 labelled muscle fibers. Red arrows point to the edge of a muscle fiber. Inset shows muscle fibers at higher magnification to better determine the size of the fiber. (**C**) Merged image showing the co-labeled white muscle fiber and nAChR in vehicle-treated animals. (**D**) α-bungarotoxin labelled nAChRs associated with white muscle fibers in THC-treated (6 mg/L) embryos. White arrow shows clusters of nAChRs. (**E**) Anti-F310 labelled muscle fibers. Red arrows point to the edge of a muscle fiber. Inset shows muscle fibers at higher magnification to better determine the size of the fiber. (**F**) Merged image showing the co-labeled white muscle fiber and nAChR in THC-treated animals. (**G**) Bar graph showing the diameter of white fibers for vehicle and THC treated embryos and (**H**) Measurement of white fiber length. *** significantly different from vehicle control, *p* < 0.001.

A similar analysis of the white fibers using the F310 antibody combined with α-bungarotoxin labelling of nAChRs provided a similar result (Figure 4). The white fiber diameter for control embryos was 7.8 ± 0.3 µm (Figure 4G), whereas it decreased to 4.6 ± 0.3 µm for THC treated embryo muscle fibers (Figure 4G, *p* < 0.05; *n* = 18–22). We did not observe any significant changes in the length of individual fibers (Figure 4H). The white fibers exhibited periodic regions of disorganization with intermittent nAChR expression (Figure 4D–F). Further, the labelling of α-bungarotoxin showed more condensed nAChR that was also disorganized (Figure 4D).

### 3.4. THC Does Not Alter nAChR Subunit Expression

**Figure 5:**
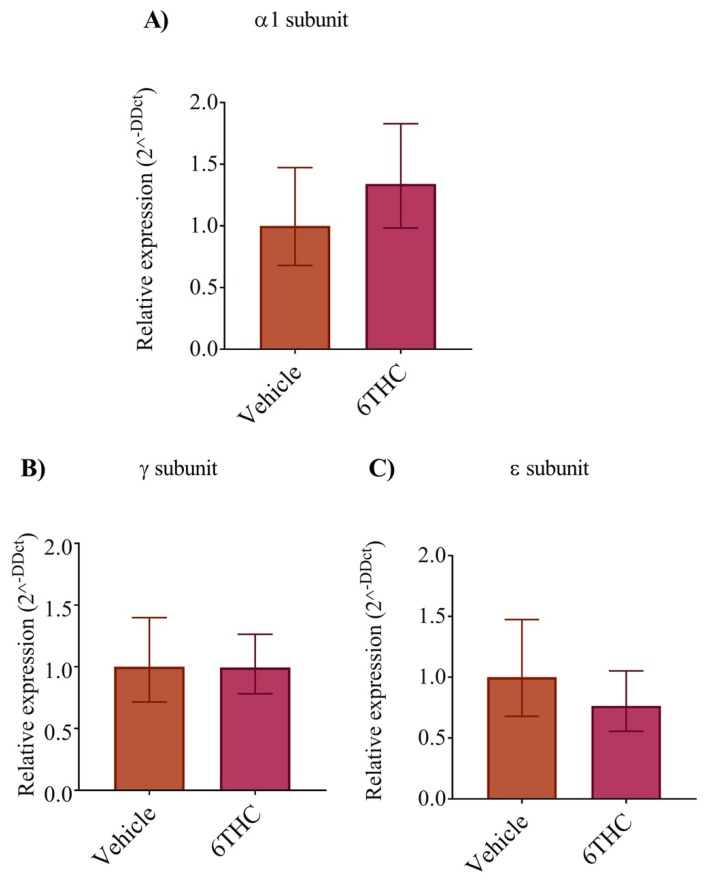
The relative levels of nAChR subunits (*α*1, *γ* and *ε*) mRNAs were analyzed by real-time qPCR. The relative expression was measured from vehicle control and THC treated embryos using expression in vehicle control as calibrator. (**A**) The relative level of α1 nAChR expression from vehicle and THC treated embryos. (**B**,**C**) The relative expression of γ and ε, respectively. Data are expressed as the mean ± SE for individual groups (*n* = 5).

To determine if the expression of the nAChR subunits was altered following THC exposure, we performed a semi quantitative analysis of the mRNA for the *α*1, *γ* and *ε* subunits in relation to the β subunit. However, we found no significant differences in the relative expression of the nAChR subunits (Figure 5A–C) suggesting that differences in nAChR subunits expression do not occur as a result of early THC exposure in our experimental paradigm.

### 3.5. THC Exposure Alters the Locomotion at 5dpf

**Figure 6:**
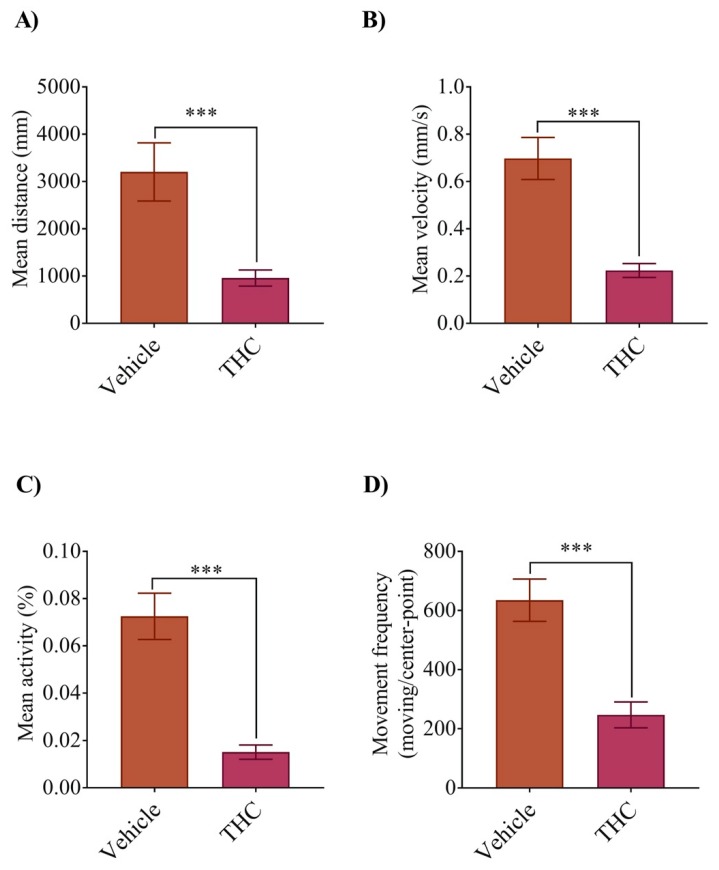
THC exposure affects free swimming activity (locomotion) of zebrafish embryos at 5 dpf. Bar graphs display changes in larval mean distance moved (**A**), mean velocity (in mm/s for one hour) (**B**), mean activity (% rate for one hour) (**C**), and frequency of swim bouts within one hour (**D**). *** significantly different from vehicle control, *p* < 0.001.

Lastly, we allowed the animals to develop until they were 5 dpf, at which age they actively swim to feed. This allowed us to determine if exposure to THC affected their basal level of activity. We found that all aspects of their movement were altered by THC treatment during gastrulation (Figure 6A–D). For instance, the mean distance swam changed from 3200 ± 620 mm/hr in the controls (*n* = 77) to 960 ± 170 mm/h in the THC treated animals (Figure 6A, *n* = 77; *p* < 0.001). The mean velocity fell from 0.70 ± 0.08 mm/s (*n* = 77) to 0.22 ± 0.03 mm/s (*n* = 79) (Figure 6B, *p* < 0.001), the mean activity fell from 0.073% (*n* = 84) to 0.015% (*n* = 84) (Figure 6C, *p* < 0.001) and the movement frequency fell from 635 ± 72 (*n* = 74) to 247 ± 44 (*n* = 74) (Figure 6D, *p* < 0.001). Taken together, these findings suggest that cannabinoid treatment during gastrulation affected neuronal morphology to a small degree, as well as the development of muscle fibers and various aspects of locomotion. These results are consistent with our previous study and suggest that developing organisms exposed to THC may experience subtle alterations in development.

## 4. Discussion

In the present study we asked whether M-cells exposed to THC at the time of their (cellular) birth (during gastrulation) experience deficits in axonal projections, or if zebrafish embryos exhibit changes in escape response properties. Multiple reports provide convincing data to show that the eCB system, particularly the CB_1_Rs, play a role in the differentiation of neural progenitor cells [31,32], the proper development of axonal projections and in neurite outgrowth [3,14,15,16,33,34]. Our findings suggest that brief exposure to THC subtly alters some aspects of M-cell morphology such as size and shape, although their axonal projections appear to be largely intact and project normally. There were minor changes in the C-start response to touch such as the angle of the C-bend. Finally, muscle fiber development was impacted to a small degree and overall activity levels were reduced.

Cannabis has been characterized as the most commonly used illicit drug in pregnant women [35] to reduce morning sickness. Moreover, in North America, there has been an increase in the cannabis use among women of reproductive age [36]. THC is the main psychoactive ingredient in cannabis and an increase in the potency and content of THC has been reported over the last 25 years [37]. Even though cannabis is used by pregnant women to reduce morning sickness there is relatively little information on the effects of cannabinoids on embryonic organisms during early development. In our research we focus on exposure to cannabinoids at some of the earliest developmental time points when the nervous system starts to form from the ectodermal tissue of the gastrula. In zebrafish, this is also the time when the M-cells first appear, around 8–9 hpf [19]. At this developmental time point, cannabinoid receptor expression is low, but mRNA coding for both CB_1_Rs and CB_2_Rs can be detected as early as the start of gastrulation [17]. In fact, in chick embryos CB_1_Rs are present from the earliest stages of neuronal life and in the developing chick they first appear in the CNS as early as the birth of the first neurons [38]. In embryonic organisms CB_1_R agonists and antagonists are capable of altering axonal growth [15], and signaling through the endocannabinoid system has been shown to play chemo-attractive and chemo-repulsive roles in developing cortex [39,40]. Several reports show an interaction between the endocannabinoid system and growth factors during early development. For instance, in cerebellar neurons CB_1_R activation linked to FGF receptor activity influences neurite outgrowth, while CB_1_R interaction with TrKB receptors in cortical interneurons is required for interneuron migration and specification [39]. Thus, the endocannabinoid system has the ability to control neuronal migration and differentiation by regulating growth factor activity. The endocannabinoid system has also been shown to modulate the expression of neurotransmitters in the basal ganglia that are involved in movement such as GABA and glutamate [7]. Others have shown that morpholino knockdown of CB_1_R in zebrafish leads to aberrant axonal growth and fasciculation of reticulospinal neurons in the hindbrain [3]. Our perturbation of the eCB system via exposure to THC did not yield similar results to these earlier studies, but we exposed animals to significantly high levels of THC so that the eCB system could be overstimulated, and we did so for only 5 h around the time of neuronal birth, whereas morpholino oligonucleotides are often functional for up to 4-5 days.

Blood plasma concentrations of THC can peak as high as 0.25 mg/L during the smoking of a single cannabis cigarette [41]. In our study, we exposed the embryos to 6 mg/L of THC while they were still in the egg casing, and it is difficult to ascertain exactly what concentration of THC equilibrates in the neuronal tissue of the gastrula. Moreover, recent analysis shows that the THC content of cannabis has increased up to 20-fold over the last 15–20 years [37,42,43]. It has been estimated that approximately 0.1%–10% of toxicants typically cross the chorion [44,45], suggesting that concentrations as low as 0.006–0.6 mg/L may be directly exposed to the embryonic neuronal tissue. Hence, we believe that the concentrations of THC (6 mg/L) used in this study may be within the physiological range experienced during cannabis use.

Cannabinoid receptors are largely localized to the plasma membrane, but they have also been shown to be associated with the endoplasmic reticulum (ER), endosomes, lysosomes, mitochochondria (mt) [17]. As a general rule CB_1_Rs are highly localized to the CNS while CB_2_Rs are mainly found outside of the CNS in systems such as the immune and digestive systems. At the subcellular level, mitochondrially expressed CB_1_Rs are found in axon terminals and dendrites of neurons. CB_2_Rs have been reported in neuronal and glial cells in the cortex, hippocampus and substantia nigra of rat brain [46,47]. In neurons, CB_2_Rs are present in the cell body and dendrites, and are therefore typically localized to presynaptic regions [46,47]. CB_2_Rs are also associated with the rough ER, Golgi apparatus and dendrites. In zebrafish, CB_1_R expression, determined by in situ hybridization, is localized to the pre-optic areas at 24 hpf and the telencephalon, tegmentum, hypothalamus and anterior hindbrain by 48 hpf [17,48]. In contrast, CB_2_R mRNA expression in zebrafish brain was relatively weak and appeared to be limited primarily to the rostral portion of the pituitary [49,50].

In this study, we investigated whether exposure to THC alters locomotion in zebrafish embryos and larvae. In particular, we set out to examine touch evoked-escape response in 2 dpf animals but also investigated locomotion in older 5 dpf larvae. While the escape response is driven by reticulospinal neuronal activity (M-cell, Mid2cm, Mid3cm neurons), swimming is generated by networks of neurons in spinal cord including excitatory & inhibitory interneurons (Ins), primary and secondary motor neurons and muscle fibers. Escape response and free swimming can be categorized as fast (>30 Hz) and slow frequency (<30 Hz) swimming respectively. Fast frequency escape responses involve the relay of sensory information to M-cells, which in turn excites a CPG network of neurons in the spinal cord that activates muscle fibers. During fast swimming, more dorsal MNs (both primary and secondary) become recruited and activated than ventral MNs. White fibers are active during fast swimming frequency but not in slow swimming. In contrast, only the most ventral MNs are active during slow swimming. The red fibers are active during slow swimming and become deactivated during faster swimming frequency. Slow frequency free swimming begins to appear at 3 dpf which last only few seconds as it consists of occasional swimming episodes. By 4 dpf, embryos exhibit beat and glide locomotion and by 5 dpf they swim more frequently. Beat-and-glide fashion consists of swim bouts, periods of rhythmic tail movement, and alternate period of rest.

The immunolabelling of muscle showed that exposure to THC resulted in smaller red and white fibers that appeared disorganized compared with vehicle controls. Zebrafish red and white trunk muscles arise from two completely separate precursor cell populations [51,52]. The red fibers are pioneer cells that migrate to the surface of the trunk where they form a single layer of muscle that becomes innervated by secondary motor neurons [52]. The white fibers develop from lateral pre-somitic cells and constitute a separate population of cells that can be identified via distinct morphological and genetic features [51]. CB_2_Rs are known to be associated with embryonic stem cells but it is unclear if cannabinoid receptors are found on muscle precursors. Cannabinoids are highly lipophilic substances and may actually remain associated with cell membranes long after the exposure time frame has elapsed. If so, then this might suggest that the effects of cannabinoids may continue long after direct exposure has ended.

In our previous study [20], we investigated the branching pattern of primary and secondary MNs involved in the CPG network. In the current study, we wanted to examine whether exposure to THC altered additional components of the network including M-cells, and then secondarily, if white and red fiber morphology was altered. Our findings show that THC exposure reduced the diameter of M-cell axons and resulted in smaller, more loosely packed, and slightly disorganized architecture of red fiber and white fiber. These findings are consistent with other studies that show that exposure to neurotoxic substances induces changes in skeletal muscle organization and composition, and disrupts the normal sarcomeric pattern, alters glycoprotein composition, and damages mitochondria [49]. While some of our findings appear to be minor, such as the small reduction in M-cell axon diameter, we believe that the key element to take note of is that a brief exposure to THC during embryological development may impact organismal growth, form, and function, and therefore, even only minor changes may have significant physiological consequences.
